# Obesity Epidemic and Its Impact on Female Fertility: Current Understanding and Future Directions

**DOI:** 10.7759/cureus.87283

**Published:** 2025-07-04

**Authors:** Radhika Malhotra, Jessica Garcia de Paredes, Alexandria Smith, Anat Chemerinski, Dhvani Doshi, Sara S Morelli

**Affiliations:** 1 Department of Internal Medicine, Rutgers University New Jersey Medical School, Newark, USA; 2 Department of Obstetrics, Gynecology and Reproductive Health, Division of Reproductive Endocrinology and Infertility, Rutgers University New Jersey Medical School, Newark, USA

**Keywords:** assisted reproductive practices, female fertility, infertility, obesity medicine, weight loss and obesity

## Abstract

Obesity is a global epidemic with profound implications for fertility in women of reproductive age. This review summarizes how obesity affects fertility by disrupting the hypothalamic-pituitary-ovarian (HPO) axis, affecting oocyte quality and reserve, and compromising endometrial receptivity. The combination of these disruptions leads to reduced fertility rates, decreased success with assisted reproductive technologies (ARTs), and increased risk for adverse pregnancy outcomes. This review also examines the impact of interventions for weight loss, including lifestyle modifications, medications, and surgery, on female fertility.

Overall, these interventions show promise in anovulatory cycles; however, their impact on live birth rates (LBRs) remains variable. Newer therapies, such as glucagon-like peptide 1 receptor agonists (GLP-1 RAs), have expanded the armamentarium of options for obesity management. Emerging data suggest potential roles for GLP-1 RAs in ovarian and endometrial function, suggesting broader applications in reproductive health. Given the complexity of obesity’s impact on reproduction, a multidisciplinary approach incorporating lifestyle modification, pharmacotherapy, and, if necessary, surgery offers the most significant potential to optimize reproductive success.

## Introduction and background

Obesity has become a global health issue, with a significant impact on reproductive health. The prevalence of obesity in reproductive-aged females in the United States was estimated to be around 40% between 2021 and 2023, according to data from the U.S. National Health and Nutrition Examination Survey (NHANES) [1]. Obesity is defined by the World Health Organization (WHO) criteria as a body mass index (BMI) of equal to or greater than 30 kg/m², which is a factor of weight compared to height [2]. With respect to race, Black and Hispanic populations in the United States are disproportionately affected by obesity - 49.6% and 43.0%, respectively - and lowest among Asians [3,4]. Obesity in women also increases risk for morbidities, including, but not limited to, hypertension, type 2 diabetes mellitus, hyperlipidemia, obstructive sleep apnea (OSA), depression, and metabolic-associated liver dysfunction [5].

As the prevalence of obesity continues to rise, its adverse effects on natural fertility and on outcomes after assisted reproductive technologies (ARTs) have become a significant concern. Not only does increasing BMI increase the risk of infertility, but increased waist circumference by 1 cm increases the infertility rate by 3% [6]. Obesity is an independent risk factor for female infertility, regardless of the presence of metabolic syndrome [7], and adversely impacts female fertility through various mechanisms, including endocrine disturbances and alterations in ovarian function and ovarian reserve [8]. The presence of obesity decreases the success rates of all available therapies for infertility, including ovarian stimulation, intrauterine insemination (IUI), and in vitro fertilization (IVF) [9]. IVF outcomes are adversely affected by obesity, with studies indicating a higher risk of complications during ovarian stimulation, and reduced fertilization and embryo implantation rates [10,11]. Once pregnancy is achieved, maternal obesity is linked to adverse pregnancy outcomes, including the risks of miscarriage, gestational diabetes, preeclampsia, cesarean delivery, and preterm birth [12-15].

In this review, we will address the pathophysiology of obesity-related infertility, review the impact of obesity on ART success rates and pregnancy outcomes, and discuss interventions to mitigate the adverse effects of obesity in women of reproductive age.

## Review

Methods

This narrative review was conducted using a PubMed search for studies evaluating the impact of obesity on female fertility, published between 1995 and 2025, using the following keywords: "fertility," "female fertility," "reproduction," "assisted reproductive technology," and "obesity." To identify studies evaluating the effect of medical and surgical interventions for obesity on female fertility, we also performed a PubMed search using the following keywords: bariatric surgery, endoscopic sleeve gastrectomy, glucagon-like peptide 1 (GLP-1) agonist, and anti-obesity medications (AOMs). Review papers that cited original references were also examined for relevant information. We included primarily human studies and referenced either animal studies or in vitro studies using animal or human cell lines when discussing pathophysiologic mechanisms. Commentaries, editorials, and trade publications were not included in this review. We excluded studies focused only on male fertility, which were considered to be outside the scope of this review. 

Impact of obesity on female fertility

Hypothalamic-Pituitary-Ovarian (HPO) Axis

**Figure 1:**
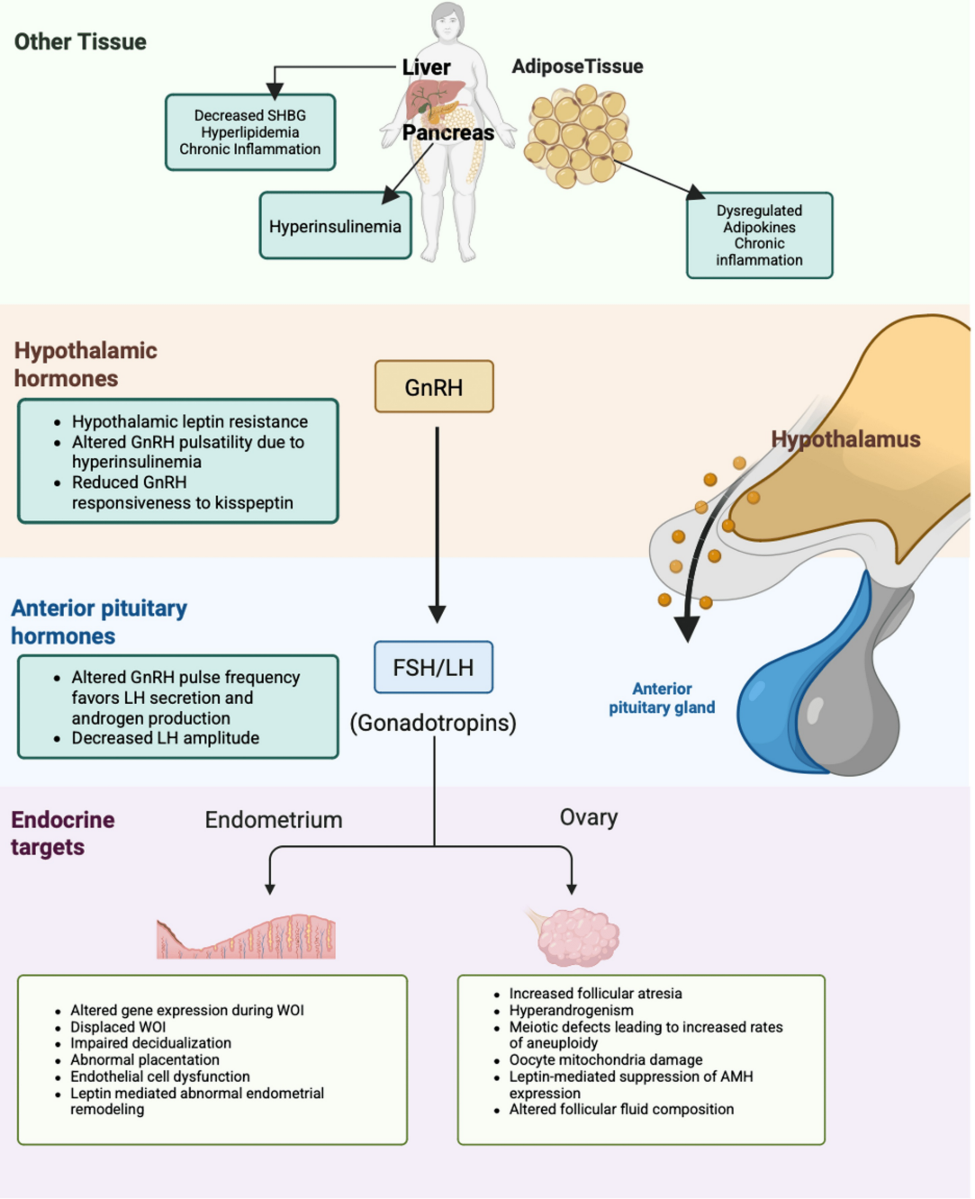
Mechanisms by which obesity affects female fertility This figure provides an overview of the adverse effects of obesity on female fertility, including mechanisms at the levels of the hypothalamus, anterior pituitary, ovaries, and endometrium. Credit: Images created with biorender.com SHBG, sex hormone binding globulin; GnRH, gonadotropin-releasing hormone; FSH, follicle-stimulating hormone; LH, luteinizing hormone; AMH, anti-Müllerian hormone

Pulsatile release of gonadotropin-releasing hormone (GnRH) is essential for the anterior pituitary release of follicle-stimulating hormone (FSH) and luteinizing hormone (LH), and subsequent ovulation [16]. Disruptions in this axis can, therefore, have significant deleterious effects on fertility. Obesity may impact the HPO axis through multiple mechanisms, including by inducing a state of leptin excess, promoting insulin resistance, and favoring a pro-inflammatory environment (Figure 1).

Obesity is associated with elevated circulating leptin, a pro-inflammatory peptide hormone involved in energy homeostasis. While GnRH neurons do not themselves express leptin receptors, populations of LepRb (Leptin Receptor, isoform b) neurons in other hypothalamic nuclei may directly and/or indirectly signal to GnRH neurons [17,18]. Mouse studies have demonstrated that obesity is associated with hypothalamic resistance to leptin signaling due to leptin receptor down-regulation, and have found that the infertility associated with obesity can be rescued by exogenous gonadotropin administration [19,20]. Taken together, these studies suggest that obesity, via elevated circulating leptin, induces a state of hypothalamic leptin resistance, with indirect effects on GnRH secretion, leading to ovulatory dysfunction and infertility.

Obesity is also characterized by insulin resistance and compensatory hyperinsulinemia, which promote hyperandrogenemia. At the central level, hyperinsulinemia alters GnRH pulse frequency, favoring increased LH over FSH secretion, which further stimulates ovarian androgen production. Peripherally, insulin suppresses hepatic synthesis of sex hormone-binding globulin (SHBG), resulting in higher levels of circulating free testosterone [12,20]. Additionally, obesity is associated with increased peripheral aromatization of androgens to estrogens, which leads to decreased GnRH and FSH release through negative feedback mechanisms [21,22]. Furthermore, the chronic low-grade inflammation associated with obesity, mediated by cytokines such as tumor necrosis factor-alpha (TNF-α), released from hypertrophic adipocytes, reduces GnRH neuron responsiveness to stimulatory inputs like kisspeptin [23]. Finally, compared to normal-weight controls, women with obesity demonstrate lower LH pulse amplitude following exogenous GnRH administration, suggesting direct pituitary effects of obesity as well. Together, these hypothalamic and pituitary-level disruptions illustrate the central neuroendocrine mechanisms by which obesity impairs female fertility [24].

Ovary

In addition to the negative impact on hypothalamic and pituitary signaling, obesity may negatively impact ovarian and oocyte function as well. Women with obesity demonstrate reduced ovarian response to gonadotropin stimulation, requiring higher gonadotropin doses and achieving lower numbers of follicles and lower estradiol levels, which may be due to reduced expression of the FSH receptor in the ovarian granulosa cells [25,26]. In a rodent study, ovaries of diet-induced obese (DIO) mice were found to have more apoptotic ovarian follicles, fewer and smaller mature oocytes, and smaller pups than the control group [27]. In mice, a high-fat diet is associated with meiotic defects, including abnormal spindle formation and chromosome misalignment, leading to higher rates of aneuploidy, while in humans, oocytes from failed fertilizations were more likely to contain abnormal spindles and misaligned chromosomes in patients with obesity compared to normal-weight controls [28,29]. Spindle formation abnormalities persist despite lifestyle modifications, indicating that there is likely some irreversible injury that occurs in the ovary due to obesity [30]. Additional evidence demonstrates that mice fed a high-fat diet have higher lipid content in the cumulus-oocyte complex and exhibit oocyte mitochondrial damage [31]. Human studies indicate that women with obesity have lower clinical and ongoing pregnancy rates following IVF, and in vitro evidence points to a mechanism involving the inhibition of granulosa cell proliferation and the promotion of apoptosis by high levels of leptin [32]. Analysis of human follicular fluid obtained at the time of oocyte retrieval has also demonstrated that elevations in free fatty acids in follicular fluid are associated with poor cumulus-oocyte complex quality [33].

In addition to its central role in mediating hypothalamic signaling, leptin treatment has also been shown to suppress in vitro granulosa cell expression of anti-Müllerian hormone (AMH), an effect, in part, mediated by the JAK2 (Janus kinase 2)/STAT3 (signal transducer and activator of transcription 3) pathway [34]. Concentrations of adipokines have been assessed in ovarian follicular fluid, and levels of TNF-α, leptin, IL-18, and IL-10 appear to be correlated with BMI, suggesting follicular fluid-specific effects of obesity [35]. Levels of adiponectin, an adipokine expressed in granulosa cells, are inversely correlated with obesity and have been linked to ovarian function and fertility. Adiponectin-knockout mice demonstrate reduced fertility compared to wild-type controls, through a reduction in ovulated oocytes and abnormal follicular development [36]. Together, these studies highlight the multiple mechanisms by which obesity (via elevated leptin, aberrant cytokine expression, and reduced adiponectin levels) impacts normal ovarian function (Figure 1).

Endometrium

Successful reproduction requires a finely orchestrated and temporally specific interaction between the maternal endometrium and the developing embryo. A key event in this process is embryo implantation, which occurs during a brief period known as the “window of implantation” (WOI). In humans, this receptive phase spans cycle days 20-23 and is predominantly regulated by the cyclical activity of ovarian hormones - estrogen, primarily estradiol (E2), and progesterone (P4) [37]. These hormones activate their respective nuclear receptors, promoting the activation and transcription of downstream genes that induce biochemical and structural changes in the endometrium. This process results in the decidualization of the endometrium, a prerequisite transformation for endometrial receptivity and subsequent embryo implantation to occur [37].

The impact of obesity on the endometrium remains an area of active investigation [8,12,15,38,39]. Evidence suggests that elevated BMI can significantly alter gene expression profiles in the endometrium during the WOI [2]. In one study, Luke et al. found distinct endometrial gene expression patterns in reproductive-aged women with obesity when compared with normal-weight controls, with differences amplified in those with infertility [15]. Other studies of human endometrium have also demonstrated altered proteomic and transcriptomic signatures in the endometrial tissue of women with obesity, indicating a potential shift of the WOI, in correlation with increasing BMI values [8]. Functionally, these molecular disturbances have downstream effects on the critical processes of decidualization and implantation [8,12,15,39]. Poor hormone response, leading to impaired endometrial decidualization, can compromise embryo-endometrial crosstalk, possibly leading to defective placentation [7,11].

In addition to its effects on the hypothalamic-pituitary (HP) axis and the ovary, in vitro models using human endometrial cell lines also suggest that leptin may play a direct role in modulating endometrial epithelial remodeling and receptivity during implantation [40]. With respect to the inflammatory milieu often present in the serum of women with obesity, increased levels of circulating pro-inflammatory cytokines and reactive oxygen species (ROS) have been implicated in endometrial endothelial dysfunction, also contributing to abnormal placental development [12,41]. These pathophysiological changes contribute to adverse pregnancy outcomes associated with maternal obesity, including hypertensive disorders of pregnancy and intrauterine fetal demise [12].

To isolate the effect of obesity on the endometrium, independent of oocyte quality, several studies have assessed IVF outcomes in recipients of donor oocytes [8,12,15,38,39]. While earlier reports suggested an adverse impact of obesity on reproductive outcomes - including decreased implantation, pregnancy, and live birth rates (LBRs), as well as higher miscarriage rates - these findings were not consistent across all studies, highlighting the complexity of the obesity clinical spectrum [37,44]. Importantly, these associations have been reported to persist even when euploid embryos are transferred, also highlighting the independent contribution of obesity to endometrial dysfunction in this population [8,12,15,38,39]. A summary of the effects of obesity on the endometrium is shown in Figure 1.

Effect of obesity on ART and pregnancy outcomes

Treatment options for women experiencing infertility include oral and/or injectable medications to stimulate ovarian follicle development, IUI, and IVF. As the prevalence of obesity continues to rise, its influence on fertility and ART has become a significant concern in both clinical and research settings. Obesity impairs both female and male fertility, impacting the success rates of ART [10-15].

The following sections will examine how obesity impacts female and male fertility, ART success rates, and pregnancy outcomes, and will discuss strategies for mitigating these effects.

Obesity and Male Fertility

Although not the primary focus of this review, it is worth mentioning that obesity has a significant impact on male reproductive health, which also compromises ART outcomes. Briefly, obesity in men is associated with altered levels of sex hormones, particularly testosterone, which is critical for sperm production [13,42]. Increased body fat leads to increased testosterone conversion to estrogen, impairing spermatogenesis [13,42]. In addition, obesity-induced insulin resistance can also impair testicular function [42].

Obesity negatively affects sperm quality, including reduced sperm count, motility, and morphology. Studies show that men with higher BMI tend to have lower sperm concentration and motility, which can reduce the chances of successful fertilization, whether naturally or through ART [13]. This reduction in sperm quality is thought to be related to increased oxidative stress and inflammation, which can contribute to sperm DNA fragmentation and impaired fertility [43].

Obesity and Fertility Treatment Success

Given the steady increase in the global prevalence of obesity, reproductive care providers are encountering an increasing number of reproductive-aged women requiring infertility care through ART [38]. The risk of infertility is calculated to be three times higher for those with a BMI of >30 kg/m², compared with those with a normal BMI [44].

A variety of factors related to obesity may impact fertility treatment outcomes. For patients undergoing IUI, the association between obesity and treatment success has been inconsistent and dependent on the type of treatment, yielding different results for oral versus injectable medication regimens [44,45]. A recent retrospective cohort study analyzing 1,959 IUI cycles of women with obesity, stratified by BMI values, concluded that elevated BMI does not reduce LBRs in the absence of ovulatory dysfunction [44]. Another retrospective cohort study analyzed 3,217 IUI cycles in 1,306 women, stratified by BMI. LBRs were similar across BMI categories, including when obesity was further broken down into WHO classes. Women with obesity had higher odds of biochemical pregnancy; however, no differences were found in clinical pregnancy, multiple gestation, or miscarriage rates when compared to women with normal BMI values. Overall, BMI was not a significant predictor of live birth [46].

Women with obesity undergoing ovarian stimulation for IVF tend to require higher starting doses of injectable gonadotropins to achieve adequate ovarian response, as well as a more extended period of stimulation, increasing the risk of complications such as ovarian hyperstimulation syndrome (OHSS) [10,11,38]. Despite the greater and longer amount of gonadotropin exposure, fewer mature oocytes retrieved and higher cycle cancellation rates have been observed in women with obesity, with an inverse association reported between BMI and ovarian reserve, as measured by AMH levels [10,38]. Overall, high BMI is associated with decreased ART success rates, including lower fertilization rates, decreased embryo quality, and reduced implantation rates [47].

There is also conflicting evidence regarding clinical pregnancy rates (CPRs) and LBR in women with obesity undergoing IVF. Some studies report reduced CPRs and LBRs, while others report no difference in outcomes compared with patients with normal BMI values [38]. A recent prospective cohort study from 2021, the Appraisal of Body Content investigation, evaluated the impact of obesity on IVF outcomes by analyzing 1,889 infertile couples [48]. The study utilized bioelectrical impedance analysis to determine body fat percentage and BMI at the time of oocyte retrieval. Although results demonstrated comparable IVF outcomes, including fertilization, progression to blastocyst stage, and euploidy rates between women with obesity and those of normal weight, the study demonstrated a statistically significant increase in the incidence of preterm delivery and very low birth weight (<1,500 g) infants, particularly when stratified by BMI.

Multiple prospective cohort studies and systematic reviews demonstrate that the number of oocytes retrieved, maturation rates, fertilization success, embryo euploidy, implantation rates, and LBRs showed no significant differences across BMI categories, contrasting with earlier retrospective studies that reported diminished outcomes in obese patients [8,11,12,38,48]. Despite studies not appearing to show a difference in euploidy/aneuploidy rates, women with obesity experience higher rates of miscarriage in pregnancies conceived via ART [12,14,38,49]. In addition, the elevated risk of perinatal and neonatal complications persists, emphasizing the need for continued lifestyle counseling and risk mitigation in this population.

Effects of Weight Loss on ART Success

Even modest weight loss before ART can improve ovarian function, egg quality, and overall outcomes after ART [50,51]. However, the effect of weight loss specifically on improving LBRs after IVF remains controversial, as multiple studies demonstrate no difference in LBRs in women who lose weight [52]. An important consideration for clinicians is that, after weight loss, adjustments in stimulation medication dosages and monitoring are essential to optimize ovarian response [53]. Decreased doses of gonadotropins might be necessary due to increased sensitivity after weight loss, and antagonist protocols are preferred to reduce the risk of OHSS [54].

Pregnancy Outcomes

Although largely outside of the scope of this review, perinatal risks and outcomes associated with obesity should be acknowledged, given that obesity is the most common comorbidity affecting women during pregnancy [55]. Miscarriage rates in women with obesity are higher compared to normal-weight controls following euploid embryo transfer [49]. Preconception obesity may be associated with fetal macrosomia [55], in part secondary to the increased prevalence of gestational diabetes in pregnancy, though the obesity-associated risk exists in non-diabetic patients as well [56,57]. Preconception obesity may, in other cases, increase the risk of fetal growth restriction and small-for-gestational-age infants, given the association with preeclampsia [56,57]. Indeed, obesity is associated with aberrant fetal growth in general, due to alterations in the nutrients and cytokines in maternal circulation, which undergo placental transfer to the fetus or directly stimulate placental secretion of growth-promoting hormones [58]. Additionally, the risk for congenital fetal abnormalities, such as neural tube, cardiac, and limb defects, increases with maternal obesity [59]. Intrapartum risks include preterm delivery, increased cesarean section rates, and anesthesia complications [14]. Beyond pregnancy, maternal obesity leads to a higher risk of childhood obesity and metabolic syndrome in offspring, creating a perpetuating cycle of continued negative health outcomes [60].

Interventions and management

The American Society for Reproductive Medicine (ASRM) recommends weight loss prior to conception in women with obesity [39]. Pre-conception weight loss leads to increased rates of spontaneous ovulation, improved response to ovulation induction agents, and a reduction in pregnancy-related complications, but has not been proven to increase LBRs after either spontaneous conception or ART [61-64]. Weight loss of 10% body weight has been associated with increased fertility, including increased ovulation and pregnancy rates [65,66].

Lifestyle modifications - diet and exercise - are recommended to all patients embarking on a weight loss journey. This includes caloric restriction and regular physical activity. The caloric deficit is key, as exercise alone has not been shown to reduce weight [67]. Nutritional deficiencies in essential micronutrients, such as iron, vitamin D, folate, and zinc, can disrupt hormonal balance and impair ovulatory function, potentially leading to infertility [68,69]. There have been extensive publications on the “fertility diet,” which encourages low-glycemic carbohydrates, dairy, and multivitamins, and decreases trans fats and protein; however, there is no evidence to suggest this diet improves conception or LBRs [70,71]. Moderate, consistent physical activity also benefits female fertility through multiple mechanisms. Aerobic exercise and resistance training are particularly effective at improving insulin sensitivity, reducing chronic inflammation, and supporting weight management [47,53]. However, excessive physical activity can be detrimental, leading to hypothalamic dysfunction and amenorrhea, especially when coupled with low body fat [72].

Pharmacologic treatments are an additional option to aid in fertility. Compared to lifestyle modifications alone, metformin has been shown to enhance weight loss effects and reduce metabolic syndrome [73]. In nondiabetic individuals with obesity, the use of metformin is associated with a mean weight loss of around 12 pounds [74]. Metformin is typically used as a weight-loss agent when there is suspected insulin resistance, as it tends to cause more weight loss in this population [74]. The ASRM recommends the use of metformin for metabolic benefits in women with polycystic ovary syndrome (PCOS) or type 2 diabetes; however, it is not a first-line agent for treating anovulatory infertility [75]. Additionally, there is limited evidence on the use of metformin for weight loss outside of PCOS, for infertility [75].

Bupropion-naltrexone is a Food and Drug Administration (FDA)-approved medication for weight loss. It functions by targeting appetite regulation, blocking the reward pathway in the brain, and controlling hunger mechanisms [76]. Contraindications include seizure disorders, anorexia/bulimia, and uncontrolled hypertension [77]. While approximately 5% weight loss is observed after 56 weeks of use, there are no studies on the direct effects of this medication on fertility [77]. Animal studies have shown increased fetal loss and developmental anomalies, leading to the recommendation that women desiring pregnancy allow for a two-week washout period prior to conceiving [77,78]. Bupropion-naltrexone is not recommended during breastfeeding, and caution should be used when continuing this medication postpartum [79].

Similarly, phentermine-topiramate functions by suppressing appetite and reducing food cravings through norepinephrine and gamma-aminobutyric acid (GABA) receptors, with approximately a 10% weight reduction over 56 weeks [79]. Given the teratogenic potential of topiramate and its risk for fetal abnormalities - such as cleft lip/palate and low birthweight neonates - contraception is recommended [80]. Limited studies exist on the use of phentermine-topiramate for pre-conception weight loss; for this reason, it is considered a suboptimal choice for women of reproductive age. Phentermine does not carry similar fetal risks; however, it has not been approved for long-term weight management. Short-term use of phentermine has been shown to induce 5% total body weight loss in women with obesity, but it was not associated with higher pregnancy rates [81]. This indicates it might be a potential solution for quick weight loss, but it does not provide fertility benefits.

Orlistat is rarely used for weight management, given the advent of newer medications. It functions by inhibiting lipase production, leading to increased unabsorbed fat but limited weight reduction [82]. Orlistat has not been proven to improve female fertility, with no differences in live births or conception even with weight loss [83]. Some studies have shown higher rates of miscarriage while using orlistat for preconception weight loss, thought to be secondary to decreased vitamin D levels and impaired fatty acid absorption [83].

Newer on the market and in popular media are the GLP-1 receptor agonists (GLP-1 RAs), such as semaglutide and tirzepatide. These are promising AOMs that have led to excellent type 2 diabetes control, lowering insulin levels and total body weight by 10%-20%. They work through numerous mechanisms to reduce weight [84,85]. GLP-1 increases the number of glucose transporters in the pancreas, allowing for a more effective insulin response. It also delays gastric emptying in the gastrointestinal tract, leading to increased satiety and decreased food intake [86]. The most common side effects associated with GLP-1 RAs are gastrointestinal, including nausea, vomiting, and diarrhea. The medication remains contraindicated in patients with a personal or family history of medullary thyroid cancer or multiple endocrine neoplasia (MEN) 2 syndrome [87]. While studies are limited in evaluating the direct impacts of these medications on fertility, the initial data are extremely promising. These limited studies have been conducted in patients with PCOS, a metabolic syndrome that is more complex than obesity alone; however, there is physiologic evidence to suggest a role for GLP-1 RAs in fertility treatment outside of PCOS. Animal studies show that the highest expression of the GLP-1 receptor (GLP-1R) is in the hypothalamus, where it regulates the pulsatile activity of GnRH and increases LH secretion [88,89]. Mouse studies also demonstrate that GLP-1 influences both estrogen and progesterone levels and increases the number of mature follicles [89,90]. GLP-1 RAs help decrease circulating androgen levels in women with PCOS; however, the exact mechanism is not well understood [91]. GLP-1Rs are also abundantly present in the human endometrium; however, their expression during the menstrual cycle remains unclear [92]. In addition to their metabolic effects, GLP-1 RAs display anti-inflammatory properties, reduce oxidative stress, and improve lipid metabolism [93].

Most of the existing studies evaluating the effects of GLP-1 RAs on female fertility have been done with liraglutide, an older GLP-1 RA that is less efficacious than semaglutide or tirzepatide [94,95]. Studies have shown that switching from metformin to liraglutide decreases androgen levels significantly in obese women with PCOS [96]. Use of GLP-1 RAs has been shown to restore ovulatory cycles in anovulatory women with obesity and PCOS and to result in more significant weight loss than lifestyle modifications or metformin alone [97-99]. Pre-conception use of liraglutide and metformin was shown to be superior to metformin alone for increasing IVF pregnancy rates and spontaneous pregnancy rates in women with PCOS who had been resistant to lifestyle treatment [100]. Importantly, GLP-1 RAs have not been adequately studied in pregnancy, and a two-month washout period is recommended prior to conception [101]. Early studies examining the use of GLP-1 RAs during pregnancy in humans have not indicated an increased risk of fetal abnormalities with first-trimester use of the medication [102-104]. A more prolonged exposure was described in a 42-year-old woman who remained on dulaglutide until 34 weeks’ gestation and delivered a healthy neonate at term without congenital abnormalities [105]. A more recent case highlighted the risk of sepsis, complicated by euglycemic diabetic ketoacidosis, in a 35-year-old pregnant woman, emphasizing the possible metabolic risks during pregnancy [106]. While there are limited reports on the use of these medications during pregnancy, they have not highlighted major congenital abnormalities. However, in the absence of prospective safety data, GLP-1 RAs remain contraindicated during pregnancy. Tirzepatide may also decrease the efficacy of oral contraceptives more than other GLP-1 RAs, and patients should be counseled regarding this risk [107].

Surgical and endobariatric interventions for weight loss can also improve fertility outcomes. Bariatric surgery requires proper patient selection, counseling to ensure patients’ understanding of surgical risks, and lifestyle modifications prior to approval [108]. Most commonly, these procedures include sleeve gastrectomy and, more recently, endoscopic sleeve procedures [108]. Patients with obesity are found to have improved insulin sensitivity and dyslipidemia after these procedures. Amenorrhea improved in up to 71% of patients who underwent bariatric surgery, proving more effective than lifestyle modifications or metformin alone, indicating that anovulation might be an indication for bariatric surgery [109-111]. Direct studies on the effect of endoscopic sleeve gastrectomy on the resumption of ovulation and LBRs do not exist; however, results can be extrapolated from similar laparoscopic procedures. Current guidelines advise against pregnancy for at least one to one and a half years post-procedure, to minimize the risk of pregnancy complications [112]. While weight is stabilizing during this time, non-oral contraceptive methods should be used due to the risk of malabsorption of oral contraceptives and essential micronutrients such as iron, calcium, vitamin B12, and folate [113].

Similarly to GLP-1 RAs, patients should be counseled that fertility may return quickly, and they should adhere to contraceptive methods until it is safe to conceive [114]. There are studies that refute this and state that early conception after weight loss procedures does not increase the risk of maternal or neonatal morbidity or mortality; however, additional robust studies are certainly needed to make practice-changing statements [115]. Furthermore, bariatric surgery prior to conception has been linked to a reduction in pregnancy-related complications, such as gestational diabetes and preeclampsia. These benefits are thought to result from improved metabolic health and more favorable endocrine profiles, which collectively contribute to enhanced reproductive outcomes [72].

## Conclusions

This review highlights the multifaceted effects of obesity on female fertility, extending beyond anovulatory infertility. Reproductive physiology is disrupted at multiple stages by obesity, involving the HPO axis, oocyte and ovarian function, endometrial receptivity, and overall pregnancy outcomes. Because of these aberrations, women with obesity have reduced fertility rates and increased perinatal risks, such as miscarriage, preeclampsia, and fetal growth abnormalities. Future research aimed at better understanding the effects of obesity on female reproduction is needed - particularly at the level of the endometrium - and targeted interventions addressing the metabolic and inflammatory milieu in women with obesity are required to develop personalized therapeutic strategies for this population. With the advent of GLP-1 RAs and newer AOMs, more research is needed on the effects of weight loss with these medications and their impact on obesity-associated infertility. This could be a breakthrough in increasing fertility and fecundity among patients of low socioeconomic status, for whom the cost of ART presents a significant barrier. Given the complexity of obesity’s impact on reproduction, a multidisciplinary approach - with lifestyle modification, pharmacotherapy, and, if indicated, surgery - offers great potential to optimize reproductive success.
